# Communication Between Patients and Healthcare Professionals in Neurological Hospitalisation: A Qualitative Photo‐Voice Study

**DOI:** 10.1111/jocn.70122

**Published:** 2025-10-09

**Authors:** Janet F. Jensen, Pernille Würtz Boehm, Liv Gølnitz Hjorhöy, Cecilie Froulund Jensen

**Affiliations:** ^1^ Department of Regional Health Research University of Southern Denmark Odense M Denmark; ^2^ Department of Neurology Zealand University Hospital Roskilde Denmark; ^3^ Department of Quality and Improvements Health Strategic Planning Region Zealand Denmark

**Keywords:** communication, healthcare, neurology, person‐centred care, quality of care

## Abstract

**Aim:**

To explore and compare patients' and healthcare professionals' experiences of communication during hospitalisation for neurological diseases.

**Background:**

Effective communication is essential for establishing strong relationships between patients and healthcare professionals, thereby ensuring patient‐centred care that respects individual values and preferences. Neurological patients face additional communication challenges due to cognitive and motor deficits, such as speech difficulties and delayed cognitive processing. Limited research has investigated how both patients and healthcare professionals experience communication in this context.

**Design:**

An explorative, qualitative design was applied within a hermeneutic framework inspired by photo‐voice methods.

**Methods:**

Data were obtained through interviews with patients (*n* = 12), one focus group discussion with healthcare professionals (*n* = 10) and six additional interviews with healthcare professionals (*n* = 6). Interviews were combined with photographs taken during the interviews. Data were analysed using reflexive thematic analysis, and the COREQ guideline was applied.

**Results:**

The analysis revealed a main theme: *The core of connected care*, encompassing three subthemes: *Guided alignment*, *A changing environment* and *Human before patient*. These themes created the foundation for effective, compassionate and humanised care. Participants metaphorically compared this to an aquarium, emphasising that, like an ecosystem, effective communication requires balance between alignment, environment and humanity. This main theme represents the quality of communication between patients with cognitive challenges and their healthcare providers.

**Conclusions:**

This study provides insight into the experiences of communication from both patients and healthcare professionals. Effective communication is important to manage treatment and engage patients in care.

**Implications for Practice:**

Improving communication, promoting shared decision‐making and enhancing the implementation of person‐centred care are key strategies for increasing patient outcomes and satisfaction.

**Patient and Public Contribution:**

None.

**Summary**

- What problem did the study address?
  ○ Effective communication is fundamental to high‐quality healthcare, shaping the patient–healthcare professional relationship, quality of care and clinical outcomes.
  ○ Communication challenges are evident in neurological care, where patients often face unique communication challenges due to cognitive impairments.
  ○ There is little understanding of the communication gaps in neurological care seen from patients' and healthcare professionals' perspectives, and how these perspectives are similar and different.
- What were the main findings?
  ○ The study highlights the importance of connected care, which evolves through a guided alignment to ensure a shared understanding, the environmental impact within a place of constant change, and humans before patients involving humanised care with empathy and structured communication to increase the quality of care despite challenges such as cognitive impairments and time constraints.
  ○ Photo‐voice captures personal insights into communication challenges in neurological care and gives a voice to patients who may have communication difficulties.
- Where and on whom will the research have an impact?
  ○ It contributes to insights on how communication strategies can create a connected care within the framework of person‐centred care based on shared understanding through a guided alignment and adaptation to a changing environment.
  ○ The insights into humanised care and guided alignment will enhance communication and care strategies for healthcare professionals, improving patient–provider interactions in hospitals and care facilities.
  ○ This could shape health policies to improve care for patients with cognitive challenges, focusing on communication, professional training and person‐centred practices.
  ○ It is relevant for clinical practice by guiding healthcare professionals in prioritising effective communication, promoting shared decision‐making, interdisciplinary teamwork and enhancing the implementation of person‐centred care in neurology units.
- What does this paper contribute to the wider global clinical community?
  ○ Communication challenges for patients with neurological diseases emphasise the importance of healthcare professionals' skillset and ethical considerations to accommodate patients' needs for patient‐centred care and involvement in shared decision‐making based on a shared understanding.
  ○ Communication is an integral part of person‐centred care; therefore, priority should be given to enhancing its implementation.
  ○ This research could shape health policies to improve care for patients with cognitive challenges, focusing on communication, professional training and person‐centred practices.

## 1 Introduction

Effective communication is fundamental to high‐quality healthcare, shaping the patient–healthcare providers' relationship, quality of care and clinical outcomes (Cypher 2019). The Institute of Medicine defines patient‐centred care as ‘*care that is respectful of and responsive to individual patient preferences, needs, and values*’, and emphasises that ‘*patient values guide all clinical decisions*’ (Barry and Edgman‐Levitan 2012). Central to this model is collaborative communication, where healthcare professionals (HCPs) engage patients in decision‐making and ensure information is accessible and meaningful (Cypher 2019; McCormack and McCance 2016). Despite the recognised importance of effective communication, patients often experience insufficient information, limited time for discussion and difficulty understanding medical terminology (Kwame and Petrucka 2021). Additionally, hierarchical dynamics in healthcare can discourage patients from voicing concerns because they fear being perceived as difficult or uncooperative. As a result, they may avoid questions, downplay symptoms or remain silent in front of multiple professionals, especially physicians (Cypher 2019). On the HCP side, time constraints and high workloads often result in rushed interactions, reducing opportunities for meaningful dialogue (Adhikari et al. 2025; Kwame and Petrucka 2021).

The consequences of poor communication in healthcare settings are well‐documented. Studies link miscommunication to delayed treatment, misdiagnosis, medication errors and even patient harm or death (Foronda et al. 2016; Howick et al. 2024). Ineffective communication also contributes to decreased patient satisfaction, increased healthcare costs and reduced adherence to treatment plans (Howick et al. 2024). These challenges become even more pronounced in neurological care, where patients often experience additional linguistic impairments. Given the complex communication challenges, a deeper understanding of both patient and HCP perspectives is essential, particularly for improving communication with patients who present mild or severe cognitive impairments.

## 2 Background

Patients with neurological diseases face unique communication challenges due to cognitive and physical impairments. Conditions such as stroke, motor neuron diseases, Parkinson's disease and dementia can impair speech, cognition and comprehension, making it harder for patients to express concerns, ask questions and engage in shared decision‐making (Paynter et al. 2019). Stroke, Parkinson's disease, amyotrophic lateral sclerosis (ALS) and traumatic brain injury are among the neurological conditions most strongly associated with communication barriers in hospital settings (Sundhedsstyrelsen 2025). For example, patients with motor neuron diseases (e.g., ALS) often experience dysarthria, a motor speech disorder, leading to misunderstandings and frustration in patient–healthcare providers' interactions (Paynter et al. 2019). Similarly, individuals with stroke‐induced aphasia struggle with both language comprehension and expression, further complicating their ability to communicate healthcare needs (Cochrane 2022). Given these challenges, shared decision‐making (SDM) is increasingly emphasised as a strategy to improve communication. SDM ensures that HCPs provide clear, tailored information, engage in active listening and incorporate patients' values and preferences into treatment decisions (Cypher 2019; National Institute for Healthcare and Care Excellence (NICE) 2021). Research shows that SDM strengthens the relationship between patients and HCPs, enhances patient satisfaction and leads to better health outcomes (van der Horst et al. 2023; Van Der Ploeg‐Dorhout et al. 2024). However, implementing SDM in neurological care is complex, as patients with cognitive impairments may struggle to process information, articulate preferences or recall past medical discussions (Foronda et al. 2016). As a result, HCPs often rely on caregivers to facilitate communication, which may unintentionally diminish patient autonomy.

While research on general patient–provider communication is extensive, studies directly comparing patients' and HCPs' experiences in neurological care remain limited. Most studies have used a quantitative approach focusing on one perspective only, leaving a limited understanding of how perspectives align or diverge (Kim et al. 2024; Olson and Windish 2010). Addressing this gap, this qualitative interview study was inspired by a photo‐voice method to capture experiences of communication challenges and facilitators from both perspectives. By combining these perspectives, we aim to provide deeper insight that can inform and improve communication practices in neurological care settings.

### 2.1 Aim

To explore and compare patients' and healthcare professionals' experiences of communication during hospitalisation for neurological diseases, inspired by a qualitative photo‐voice method.

## 3 Methods

### 3.1 Design

An explorative, qualitative design was applied within a hermeneutic framework, inspired by photo‐voice methods and analysed using reflexive thematic analysis.

### 3.2 Methodological Framework and Photo‐Voice

The hermeneutic philosophical framework provided the overarching interpretive stance (Gadamer 2013). Hermeneutics emphasises that understanding is always situated, shaped by the interpreter's preunderstandings and developed through a dialogical movement between parts and wholes, often referred to as the hermeneutic circle (Gadamer 2013). The hermeneutical framework included preconceptions, preunderstandings and prejudgments about communication in a neurological hospital setting, which were reflected on and applied throughout the process, aiming to deepen the understanding of the participants' experience during the interpretive process (Gadamer 2013). This approach aligned with the study's aim of exploring communication experiences in a neurological hospital setting, particularly among participants with potential cognitive or linguistic impairments, where interpretation of meaning extends beyond descriptive categorisation.

The photo‐voice method is a participatory approach that combines photography with interviews or group discussions. It is grounded in the principle that visual methods can empower participants to represent aspects of their lives that may be difficult to capture through words alone (Wang and Burris 1997). Wang and Burris (1997) outlined a six‐step process for conducting photo‐voice research: (1) identify the issue and recruit participants; (2) provide training and address ethics; (3) generate images taken by participants; (4) facilitate discussion and reflection as a basis for dialogue; (5) analyse and interpret in collaboration with participants and researchers and (6) disseminate findings (Wang and Burris 1997). In this study, the steps were adapted to accommodate participants with cognitive and linguistic challenges and to support their ability to articulate experiences more freely. Photographs were taken with patients during interviews, interpretations were discussed with participants in the same setting, and the analysis was subsequently conducted by the researchers. Interpretation and analysis are commonly conducted collaboratively between participants and researchers, with images serving as ‘triggers’ for reflection, storytelling and meaning making (Catalani and Minkler 2010; Harper 2012; Sitvast et al. 2008; Wang and Burris 1997). In this study, participants were invited to take photographs illustrating their experiences of communication during hospitalisation. The photographs were used as elicitation tools to stimulate recall, clarify intentions and support reflection during interviews, thereby enriching the depth and authenticity of the narratives (Catalani and Minkler 2010; Sitvast et al. 2008). They complemented verbal communication, providing alternative ways to articulate emotional, embodied and contextual dimensions of hospitalisation that might otherwise remain unspoken (Catalani and Minkler 2010; Sitvast et al. 2008). The method was particularly suited to this study population because it enables participants to express experiences that may be difficult to convey verbally, especially for individuals with cognitive or linguistic challenges such as aphasia (Harper 2012; Wang and Burris 1997).

### 3.3 Setting, Sampling and Data Collection

**FIGURE 1:**
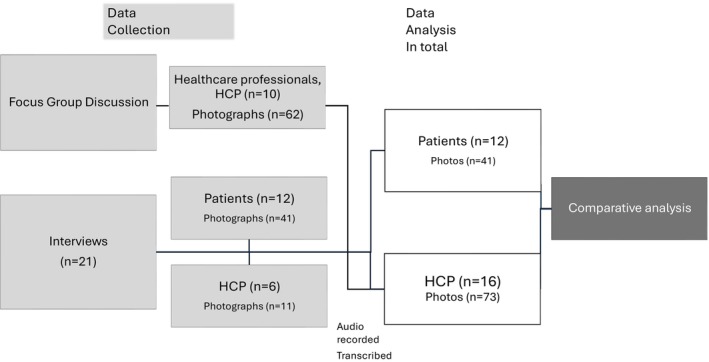
Data collection process. [Colour figure can be viewed at wileyonlinelibrary.com]

The setting was a Danish acute neurological hospital department with approximately 6500 annual admissions, including a stroke unit and a general neurology ward. The data collection took place from December 2022 to February 2023. The study participants consisted of two groups: patients (*n* = 12) and HCPs (*n* = 16). Patients were admitted to the Department of Neurology at Zealand University Hospital, Roskilde, Denmark and HCPs were affiliated with the department, Figure 1.

The sampling strategy for patients was maximum variation in age, gender, diagnosis and length of hospital stay. Maximum variation was used to capture both positive and negative experiences with communication during hospitalisation from patients. The inclusion criteria for patients were: (1) admitted to the neurological department; (2) ≥ 18 years of age and (3) gave written informed consent. Exclusion criteria were if patients had delirium, were unconscious or had severe aphasia that prevented consent. Patients' recruitment took place during hospitalisation. Following the focus group interviews with HCPs, the researchers contacted the two ward nurse managers who acted as gatekeepers in identifying eligible patients according to the inclusion and exclusion criteria. Once identified, the researchers approached the patients in person, provided both verbal and written information about the study and invited them to participate. Interviews were conducted subsequently, either the same day or the next, depending on patient condition and preferences (*n* = 12). For the recruitment of HCPs, a criterion sampling strategy was used with the following criteria: (1) Affiliated to different units in the neurological department (i.e., two hospital wards, an ambulatory setting and the centre for somatoform disorders); (2) Different professional backgrounds and (3) availability to participate during work hours. HCPs were recruited in two phases. In the first phase, management leaders identified and invited participants to a focus group discussion based on their roles and perspectives to ensure broad representation across different staff groups and units within the department (*n* = 10). The researchers prepared formal invitations, which were then distributed by department managers to avoid any sense of obligation or pressure to participate. HCPs were contacted via internal email, emphasising that participation was voluntary. In the second phase, a smaller number of HCPs, who did not participate in the focus group discussion, were selected by the research team and invited to participate in individual follow‐up interviews (*n* = 6). The selection was based on participants' willingness to further elaborate on themes that had emerged during the focus group discussion. This allowed for a deeper understanding of specific topics by exploring them from an individual perspective. These HCPs were contacted directly in person by the researchers, and interviews were scheduled during regular working hours at the participants' workplace. In total, 16 HCPs participated: 10 in the focus group interview and 6 in individual interviews. All interviews and focus groups were conducted by the research team. Before participation, both patient and HCP participants received verbal and written study information, and written informed consent was obtained from all. No participants declined to participate or dropped out.

The interview guide included two open questions: ‘What are your experiences with communication (with staff or patients)?’ and ‘What is most important for you in communication (with staff or patients)?’ Probes and pauses were used during the interviews to validate statements from participants (Malterud 2011). After the focus group interview, an initial coding of the issues discussed was sent via email to HCP for an accuracy check. Interviews were audio‐recorded and transcribed by the first author. The focus group interview lasted 58 min, while individual interviews had a mean duration of 22 min (range: 7–43), with HCP interviews generally being shorter (range: 7–21).

Participants and interviewers took photographs during all interviews to support and illustrate verbal statements (Harper 2012). Participants chose what to photograph based on what they found most important to illustrate their narratives. If a participant was unable to operate the camera, the interviewer took the photograph under the participant's direction (Harper 2012). All photos were taken with a digital camera. The final sample photographs from patients were *n* = 41, and HCP were *n* = 73.

### 3.4 Data Analysis

Data were analysed using reflexive thematic analysis (Braun and Clarke 2021). This approach aligns with an interpretive orientation and moves beyond descriptive categorisation to consider meaning and context (Braun and Clarke 2021; Wang and Burris 1997). Textual and visual data were analysed together in an iterative process moving between photographs, the interview narratives and the broader thematic structure (Braun and Clarke 2021; Gadamer 2013; Wang and Burris 1997). This approach allowed for a more nuanced understanding of participants' perspectives on communication during hospitalisation.

All interviews were facilitated by an experienced moderator accompanied by an observer. The moderator and observer took field notes during the interviews (Malterud 2011). Focus group interviews were conducted by the first and third authors, and individual interviews by the first and second authors. Reflection notes were completed after each interview to assess data saturation, which was reached when no new information emerged. In addition to the interview transcripts, participants' photographs were included in the analysis. The analysis followed Braun and Clarke's six‐step process: familiarisation, coding, generating initial themes, developing and reviewing themes, defining and naming themes and writing up (Braun and Clarke 2021). We used investigator triangulation within the research group, including mutual discussions. This approach attempts to identify, analyse and report themes within the gathered qualitative data (Braun and Clarke 2021). For comparison of the patients' and HCPs' experiences, we performed two analyses: One for the patients and one for the HCPs. In line with hermeneutic principles, both photographs and narratives were understood as forms of expression that contributed to the meaning of participants' experiences of communication (Gadamer 2013). While the photographs were not analysed as separate visual data, they were viewed as support for the interpretive texts. Photographs contributed meaningfully to the development of themes by enriching the interpretation of the verbal accounts, Table S1. The analysis began with narrative listening, supported by field notes and interview summaries, to initiate the thematic analysis. The next step was to search for sorting codes and categorise codes into themes. These themes were then revised and sorted into distinct themes with corresponding subthemes. The critical statements were sought in the collected data material about the participants' experiences of communication. The first and last authors completed the reflexive thematic analysis. Finally, the themes from the two analyses were used in a comparative analysis to understand differences and similarities in the experiences of communication between patients and HCPs. The consolidated criteria for reporting qualitative research (COREQ) guideline was applied (File S1) (Tong et al. 2007).

### 3.5 Ethical Considerations

The Helsinki Declaration was followed, and participants were included after oral and written informed consent had been obtained. Informed consent required that participants were oriented to time, place and person and free of delirium at inclusion. For those with cognitive impairments, additional safeguards were applied, such as simplified consent forms, verbal confirmation and involvement of relatives, to ensure comprehension and voluntary participation. Participation was voluntary and confidential, with no impact on treatment or employment, and could be withdrawn at any time. Photos depicted anonymous objects and unidentifiable people. However, photos should not capture private moments (e.g., end‐of‐life discussions) due to ethical constraints. The National Committee on Health Research Ethics was not required in this qualitative study (Not subject to ethical committee review, no. EMN‐2023‐02212), but the local Danish Data Protection Agency (EMN‐2023‐04115) approved the study. All participants gave written consent to participate in this study. They were informed that participation in this study was confidential and did not have any influence on patients' treatment or HCPs' work or terms of employment.

### 3.6 Rigour and Reflexivity

Reflexivity involves recognising factors influencing the research process (Guba and Lincoln 2005; Malterud 2001). In this study, investigator triangulation was employed, where investigators engaged in discussions during the analysis phase to question and expand the interpretation of data and derived themes to supplement and challenge each other's insights (Guba and Lincoln 2005; Malterud 2001). These discussions, combined with the author's knowledge of neurology departments, treatment and care, ensured credibility. Transferability was achieved through detailed descriptions of the context, methods, intervention, implementation and evaluation processes, enabling the findings to be applied to similar settings or contexts. Dependability was enhanced by including participant quotes, and transparency was improved by clearly describing the sampling, data collection and analysis processes (Malterud 2001). Confirmability was established by using NVivo with logs, reflective notes kept by the first author during the study, and researcher triangulation, which expanded the interpretation of data and the development of themes (Malterud 2001). The research team was multidisciplinary, comprising experts from nursing and sociological fields with both qualitative and quantitative research experience.

## 4 Findings

### 4.1 Participant Characteristics

The participants were patients (*n* = 12) and HCPs (*n* = 14). All participants were recruited from the Department of Neurology at Zealand University Hospital, Roskilde, Denmark. Patients' mean years of age was 60 (range: 34–85), and 11 patients had at least one other comorbidity. Of patients, 10 were hospitalised between 1 and 65 days (mean: 13.6 days), and two were affiliated with ambulatory care facilities. Additionally, 4 out of 12 patients had mild language difficulties, such as mild expressive aphasia (Table 1).

**TABLE 1:** Patients characteristics.

Patients	Sex	Age	Marital status	Children	Education	Diagnosis	Comorbidities	Language difficulties	Length of stay^a^	
P1	F	82	Widow	2	Less than high school	Parkinson	7	Yes	H	13
P2	Male	76	Single	0	Vocational education	Tumour Cerebra	4	No	H	12
P3	F	35	Cohabitating	2	Military	Bodily Distress Syndrome	3	No	H	18
P4	F	40	Cohabitating	3	Less than high school	Sclerosis	3	No	A	729
P5	F	38	Single	3	College	Stroke	1	No	H	1
P6	Male	85	Widow	2	Less than high school	Stroke	6	Yes	H	2
P7	Male	68	Single	0	Less than high school	Tumour Cerebra	8	Yes	H	6
P8	Male	83	Married	1	Vocational education	Guillain–Barré syndrome	5	Yes	H	65
P9	F	34	Cohabitating	1	College	Migraine	1	No	A	182
P10	Male	61	Single	0	Military	Sclerosis	2	No	H	2
P11	Male	55	Married	2	Less than high school	Stroke	1	No	H	3
P12	F	64	Single	2	College	Guillain–Barré syndrome	0	No	H	14

^a^ Length of stay measured in days at hospital (H) or ambulatory care facilities (A).

HCPs mean years of age was 37, ranging from 23 to 61 years, and had worked in the field of Neurology between 6 months and 26 years. They held various positions in the department (Table 2).

**TABLE 2:** Healthcare professionals' characteristics.

HCPs	# of participants	Sex (male)^a^	Age^b^	Work experience in neurology^b^	Employed in the department^b^	Position
FGD*	HCP 1–10	10%	37,4 (23–61)	8 (½‐26)	7 (½‐10)	Physician (*n* = 1) Nurse (*n* = 5) Nurses' assistant (*n* = 2) Neurophysiologists (*n* = 1) Secretary (*n* = 1)
Interviews	HCP 11–16	25%	39 (28–56)	13 (2–25)		Physician (*n* = 1) Nurse (*n* = 4) Secretary (*n* = 1)

^a^ Percentages (%).

^b^ Years (mean, range).

### 4.2 Photographs

The photographs depicted themes from the perspectives of patients and HCPs; some were illustrations of the environment and objects, while others were close‐ups of body parts. For patients, ‛guided alignment’ was shown as an ear listening and having loved ones to support understanding. HCPs illustrated ‘guided alignment’ as communication across different platforms: In the medical record; on the e‐health platform; and with HCPs sitting at a table or by the bed. The ‛changing environment’ was depicted by patients as the hospital surroundings, especially their patient room, which contributed to shaping the communication dynamics. The ‛changing environment’ was displayed by HCPs as the work environment in different settings within the hospital, such as ambulatory care facilities, the ward, hallways or the physician's office with other HCPs. Patients presented the theme ‘human before patients’ as a chair beside the bed and personal items in their room, such as a book, letters or family pictures. This illustrated the importance of meeting individuals where they are, with attentiveness, openness and curiosity, while recognising and respecting their unique personalities. HCPs illustrated ‛human before patient’ with notes on paper. This may indicate an effort to prioritise the person behind the diagnosis by enhancing memory retention, gaining deeper engagement with patient narratives and promoting more thoughtful decision‐making. But it can also indicate a task list to ensure that no symptoms of the patient are forgotten. These themes highlighted various aspects of ‛the core of connected care’.

### 4.3 Main Theme: The Core of Connected Care

**FIGURE 2:**
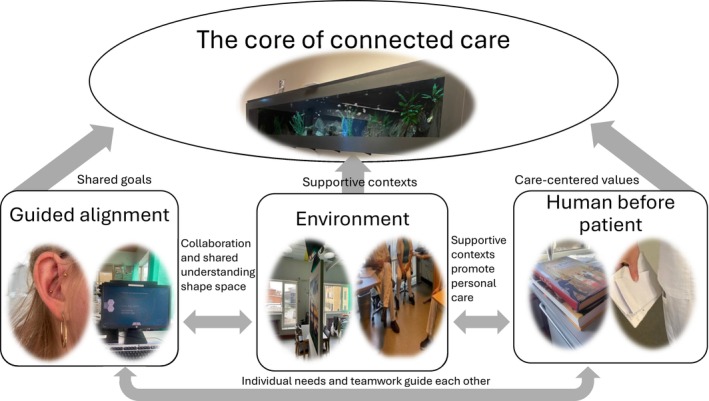
The analytical process. [Colour figure can be viewed at wileyonlinelibrary.com]

The main theme that emerged, shaping the meaning of communication experiences during hospitalisation in a neurological department, was ‘*The core of connected care’*. This main theme represents the quality of communication between patients with cognitive challenges and their healthcare providers and is metaphorically illustrated by the participants as a picture of an aquarium. This reflects the balance needed for meaningful communication in a neurological department. The main theme was linked to the three themes: guided alignment, emphasising shared understanding between patients and HCPs; the changing environment (a place for constant change), highlighting the necessity for flexibility in an ever‐changing hospital setting; and the human before the patient, prioritising empathy and recognising the person beyond the condition. Guided alignment illustrates respect for patient preferences and emphasises shared understanding through information exchange and mutual agreement. The changing environment relates to creating supportive care contexts and establishing conditions that enable informed choice. Human before patient aligns with a focus on emotional support, dignity and recognition of the person beyond the illness, which, in turn, reinforces the importance of acknowledging the patient's perspective and values within decision‐making processes. The themes were closely interconnected, mutually influencing and reinforcing one another in practice. Guided alignment and changing environment shaped each other through collaboration and a shared understanding of care priorities. Changing environment and Human before patient were linked in that supportive settings promoted personalised, individualised care. Similarly, Human before patient and Guided alignment were interdependent: individual needs informed teamwork and shared decision‐making, while teamwork ensured that care remained person‐centred. Collectively, the three themes converged within the core of connected care, forming a dynamic and reciprocal relationship that provided the fundamental support for effective, compassionate and humanised care. During interviews, participants referred to the actual aquarium located in the department, using it as a metaphor in which diverse ‘fish’ (individuals) must thrive in harmony within their shared environment (Figure 2). Selected quotations are presented in the text differentiating patients and HCPs.

### 4.4 Theme I: Guided Alignment

Guided alignment was the first theme that evolved from the interviews, covering patients' and HCPs' perspectives. Guided alignment illustrates individualised care and a process of mutual agreement, as both patients and HCPs sought to cocreate understanding and align on care decisions.

#### 4.4.1 Patient's Perspectives

For patients, guided alignment symbolised that they had been on the same page in achieving a shared understanding, which allowed for optimal communication based on their needs. The shared understanding required collaboration between patients and different HCPs. Having different HCPs meant that some patients had to repeat and explain their situation several times, challenging cognitive capacities and disrupting the trust‐building process.

I trust that's how it is. So, when a stranger [the night‐shift nurse] comes in late in the evening and kind of puts those thoughts in my head, making me feel like I have to explain how and why, and it's someone I don't even know (P3).

However, meeting different HCPs did not always negatively impact communication. It depended on the HCPs' attitude. When they were kind, compassionate, attentive and informative, it made patients feel safe and well‐cared for. Humour was perceived as an icebreaker to lighten the tone and ease the severity of the conversations. The ability to express their thoughts and needs strengthened relationships and made patients feel important and valued, which signalled authenticity. Patients considered communication effective when it addressed one step at a time, avoiding confusion from multiple questions or simultaneous tasks. This secured clear communication, leaving no room for misunderstanding. Effective communication also included shared decision‐making in treatment choices, integrating patient preferences and values while remaining open to expert opinions. In contrast, delayed responses or lack of communication were described as major obstacles for trust. Trust was built on open and direct communication.

Tell me what's wrong. I'm not the type to sit and self‐diagnose on the internet. I need it laid out straight in front of me, and then I'll deal with it. Then I'll deal with it (P10).

Neurological symptoms influenced patients' cognitive abilities (cognitive fatigue, language and memory difficulties). This required them to prioritise energy, find the right words and support memory when meeting many HCPs. Ineffective communication was often described as a lack of understanding, caused by unfamiliar medical terms, medical jargon or implicit language. Patients relied on HCPs for information on prognosis, treatment and plans. Misalignment happened in situations with sudden changes or deterioration in illness, where HCPs had to rush the medical procedures. Other patients spoke of misalignment due to prolonged waiting times, staff distractions or lack of follow‐up. The involvement of multiple HCPs (physiotherapists, neuropsychologists, doctors and nurses) complicated knowledge sharing across HCPs and with patients. As a result, some patients took the initiative to manage their treatment and rehabilitation and relied on their own expertise to secure continuity.

I saw that the stationary bike was there, and it definitely could use a bit of movement. Then the physiotherapist said, ‘We should be able to figure that out’. And of course, the great thing about all these mobile phones is that they just called after her. Then she was gone again, and she didn't come back (P2).

#### 4.4.2 HCP's Perspectives

For HCPs, guided alignment covered a shared understanding through professional skills and ethical considerations. Patient narratives were seen as central to diagnosis and treatment planning. However, time constraints often forced prioritisation of clinical tasks over dialogue, affecting patients' experience of quality. A key tension lay between professional quality and patient‐perceived quality. For example, rehearing a patient's story supported clinical reasoning but could be misinterpreted as inefficient. When such practices are not clearly explained, they risk being perceived by patients as inefficient or lacking engagement. Time pressure challenged the opportunities for dialogue, compromising the patient experience and causing a sense of professional conflict as they attempt to balance task prioritisation with their commitment to delivering high‐quality, person‐centred care.

It's somewhat a prioritization between patient‐perceived quality and professional quality. If we were better at communicating about why we do certain things, patients would better understand that there is a professional basis behind it, not just because I don't feel like reading the medical record, but because I want to hear the story again. Medical history is crucial, and I want to hear you tell it in your own words so I can understand it in a different way (FGD, Physician).

Professional skills covered empathy, respect, equal communication and being approachable. Some HCPs emphasised first impressions, with proper introductions as essential. Missing communicative guidelines influenced their approach, leading to varied and individual practices. Many had to take the initiative to obtain patient information from colleagues.

It becomes individual how things are done. So, if you could have some guidelines for what the process looks like in these transitions between these different phases of the diagnostic process and so on, it would help ensure the patient is better followed up. But it would also benefit the staff, as they would have a clearer understanding of what needs to happen, when, and so on (FGD, Neuropsychologist).

HCPs strongly considered the ethical approach in their communication, including the location of conversations. Department layout often necessitated discussions in shared rooms or hallways, leaving HCPs hesitant to ask sensitive questions. Physicians and therapists have a rigid checklist for achieving patient narratives but decided to postpone sensitive questions due to the presence of others. Few nurses described completely leaving out sensitive questions. Multiple patients share rooms, and some are even located in the hallway, which makes private physical examinations difficult. Lack of privacy was described as uncomfortable and sometimes undignified, especially during admissions, acute situations, end‐of‐life care or when delivering bad prognoses.

It is extremely uncomfortable to have a consultation in the middle of the hallway, unfortunately those are just the conditions, because there is often patient overcrowding. It is intrusive to talk to someone (in the hallway), after all, it doesn't concern anyone else (Interview, Physician).

HCPs identified ineffective communication systems and interruptions as major barriers. The main cause of ineffective communication was the use of multiple communication systems. Multiple electronic systems across hospitals and healthcare sectors hindered continuity and collaboration. This lack of continuity results in incomplete information, ultimately preventing the establishment of a shared understanding between patients and HCPs. Interruptions during consultations further reduce patient understanding. Limited patient knowledge, language barriers and medical jargon also complicated shared understanding.

### 4.5 Theme II: Changing Environment

The changing environment in the hospital was perceived differently by patients and HCPs. Patients' perspectives focused on the hospital environment, while HCPs focused on the work environment. Both described it as constantly changing. This reflected how environmental stability, or instability, could support or hinder patient participation and communication.

#### 4.5.1 Patient's Perspectives

The hospital environment played a significant role for patients in shaping communication dynamics, influencing how, with whom and where interactions took place. One patient viewed the bell rope as a safety line, noting its location and function, and considered it a crucial aid. Open access to electronic medical records was viewed as ambivalent. For some, it was an opportunity to clarify their understanding of their condition and symptoms, or to correct misunderstandings about their pre‐existing health, comorbidities and medications. However, others found the records confusing and preferred to call the hospital or receive letters in their electronic mailbox instead.

I have called a few times, and it was good to talk to the nurses; otherwise, communication is through e‐Boks and my health platform (P9). I can't read that language. You can have all the e‐health records you want, but it's all gobbledygook for non‐professionals (P2).

Patients also mentioned difficulty in distinguishing between HCPs, as they all appeared the same. Name badges were often turned around or too small to read. They appreciated having a designated contact HCP, as it meant they limited interaction with multiple HCPs. Additionally, many patients valued the opportunity to talk with fellow patients about nonmedical topics. Several expressed concerns about transitioning home and poor cross‐sector communication. For one patient in the ambulatory care facility, the hospital felt like a peaceful place—A home away from home. Other patients described the environment as distressing due to constant noise, lack of privacy and unpredictability. These stressors demanded considerable patience, which some found difficult to maintain, partly due to their neurological condition. Experiences of reduced frustration tolerance and sensitivity to delays suggested that neurological changes affected emotional regulation. A sense of being forgotten or deprioritised by busy staff further contributed to feelings of invisibility despite recognising the staff's kindness and workload. Together, these accounts highlight how environmental and neurological symptoms intersect in shaping patients' emotional responses and perceptions of care.

I hate it the most when they (HCPs) say 'two seconds'. With the tumour I have in my brain, I think it affects my patience, and I don't have much patience (P7).

#### 4.5.2 HCP's Perspectives

HCPs perceived the changing environment as their workplace, shaped by designated spaces, professional communication and collaboration. Communication occurred throughout the hospital ward. Team discussions mainly occurred during morning conferences or at team meetings without patients. Patient conversations usually took place in their rooms. HCPs experienced calm, private settings that enhanced communication, whereas shared rooms with noise and interruptions hindered understanding. Although the content remained the same, a peaceful setting improved comprehension. Effective communication required clear, consistent dialogue and the use of multiple methods (e.g., verbal, written and digital tools). Some emphasised tailoring time, pace and information to the individual. Sharing expertise across disciplines, for example, speech therapists using visual aids, improved communication and efficiency.

From an interdisciplinary perspective, we utilize each other's expertise and ensure that our knowledge within our respective fields is communicated. It is relevant for other professional groups, allowing us to optimize patient pathway (FGD, Nurse).

HCPs also reflected on equality in the relationship with patients and relatives, acknowledging that professional expertise could create power imbalances. Addressing this dynamic was seen as essential for trust and mutual respect.

These are vulnerable patients and relatives we are dealing with. There may be a lack of equality in the sense that some of us have a tendency to place ourselves above patients and relatives because we know a great deal that they do not know (FDG, Nurse).

HCPs identified systemic barriers that hindered person‐centred care. Staff shortages, overcrowded wards and high patient loads created pressure and limited time for meaningful communication or shared decision‐making. Communication was further challenged by difficulties in reaching the right person at the right time, disrupted rounds and mismatched schedules between physicians and nurses. Participants suggested that structured interdisciplinary meetings with dedicated time could enhance coordination, create a more cohesive workflow and improve the continuity of patient care. Effective collaboration depended not only on organisational planning but also on workplace culture, where permanent staff provided continuity, and openness to feedback was valued.

It is important to follow the norms on when, where, and how to talk to patients and colleagues. I believe it requires permanent staff, expertise, and self‐awareness. Sometimes, people lack insight into how to talk about others or how they'd like to be addressed. It also requires the ability to reflect and accept constructive criticism (Interview, Physician).

### 4.6 Theme III: Human Before Patient

The concept of ‘human before patient’ was perceived differently by patients and HCPs. Patients strongly emphasised dignity, compassion and recognition beyond the diagnosis, while HCPs gave it less attention. It illustrated how shared decisions relied on mutual understanding.

#### 4.6.1 Patient's Perspectives

Patients perceived their story as fragmented when their values and personal approaches to managing chronic disease(s) were not incorporated into their hospital treatment. This could be alleviated by HCPs treating them as individuals rather than merely as patients. Positive experiences occurred when patients felt respected, their dignity acknowledged and they were met with empathy and understanding. Simple acts could create honesty and mutual respect, such as sitting at eye level, giving a warm proper greeting or small talk about personal items in the room. For most, informal and light‐hearted exchanges with HCPs created a sense of familiarity and safety. Such interactions contrasted with strictly task‐focused communication, which could feel impersonal. The familiarity reinforced trust and comfort, supporting emotional support, dignity and relationship‐building.

We joke around a bit with each other, and there has to be room for that. That's something I really like, because it makes me feel safe, at least it does for me. I like that it feels a bit familiar (P4).

Patients experienced that having a personal connection with HCPs added warmth and reassurance. Presenting themselves in a dignified manner contributed to confidence, self‐respect and self‐worth. They valued attentiveness and presence, ensuring they did not feel like a burden. They also appreciated support for maintaining autonomy, which assisted them in feeling in control.

Being able to take care of yourself ‐ then things are going well! (P6).

Living with a chronic disease(s) is a journey of challenges, uncertainties and adjustments. Patients often felt unsure about their diagnosis, prognosis and the future at home. Vague symptoms and overlap led to additional examinations and unanswered questions. Over time, many patients recognised patterns in symptoms and learned to understand their condition. Accepting disease meant recognising life would not be the same as before. A diagnosis could provide clarity and validity. Hospitalisation was often overwhelming due to limitations, fear of consequences and unpredictability. Patients also worried about loved ones and not being a burden. To manage, many patients focused on 1 day at a time, engaging in care planning and staying active to maintain strength. Using a calendar helped with remembering appointments, while clear, step‐by‐step explanations during medical procedures could alleviate fears. Ultimately, living with a chronic disease required adapting, reducing anxiety and regaining a sense of daily control.

I don't know if I know things others don't think about, but for me it is normal to be on the other side of these things, all this equipment, examinations and so on. It is my world (P5).

#### 4.6.2 HCP's Perspectives

Few HCPs mentioned the importance of viewing patients as individuals rather than merely a diagnosis or a task. New patients were often introduced via written summaries, which risked reducing them to a list of tasks rather than recognising their personhood. HCPs valued patience and time to fully understand the individual behind the diagnosis but described how this was rarely prioritised. Interactions were often limited to medications, procedures or examinations. This routine approach, repeated across groups of HCPs within the system, reinforced a cycle of dehumanisation. In contrast, when time was prioritised, HCPs could form a more comprehensive, human‐centred understanding, supporting care that recognised the person beyond the disease.

Time is rarely spent on that (information sharing)—it often becomes ‘Let me borrow your arm and measure your vitals’. Again, we forget to see the person behind the patient, and this happens repeatedly in groups within the system. It has become routine for us, and that is the core of the problem (FGD, Nurse).

Human before patient highlighted that quality care relied on an adaptable, responsive approach tailored to each person's needs and preferences, focusing on their humanity rather than condition. This required skilled communication to adapt without losing continuity. While it made care more person‐centred, it also posed challenges in balancing flexibility with equitable standards. HCPs noted that without transparent communication platforms, continuity and building on colleagues' work were difficult, limiting coherent, person‐centred care.

I consider quality for the patient, which can be like the way the wind blows, meaning very individualized (FGD, Nurse). … We need a communication platform so that I can go in and continue the other person's work. I don't think it's transparent what the other person has left behind, and I find that problematic (FGD, Nurse).

### 4.7 Comparison of Communication

Patients and HCPs spoke of a professional skill set about values and effective personal qualities that could enhance a shared understanding. Patients specifically mentioned the importance of humour in their interactions. Patients' ability to share their narratives was influenced by cognitive capacity, while for HCPs this process was affected by interruptions that reduced patients' understanding of their condition and care plan. Few patients described having a limited understanding of their condition but accepted the unpredictable hospital situation and relied on HCPs to be informative about prognosis, treatment and plans. Both groups described that medical terminology hindered shared understanding. HCPs also regarded ethical considerations, which were not a focus for patients. Patients viewed the hospital as a safety line, but since all HCPs appeared similar, having a designated contact person was appreciated. Patients adapted to the changing environment, while HCPs regarded familiarity with the patient as central to the relationship. Patients and HCPs took initiative in different ways: patients managed their conditions day‐to‐day and benefited from step‐by‐step, context‐specific explanations tailored to their circumstances. HCPs gathered information through various sources, methods and documentation systems. Both groups experienced the hospital environment as busy and sometimes stressful. Simple acts of kindness and authentic presence were highly valued by patients. HCPs also expressed the importance of connecting with the person behind the patient. Maintaining this focus required constant attention, but system demands and structure often diverted their focus, risking loss of the human aspect.

While both patients and HCPs valued clear, respectful communication and maintaining a human connection, their emphasis differed. Patients prioritised recognition as individuals and valued relational aspects such as humour, familiarity and attentiveness, whereas HCPs focused more on professional processes, clinical priorities and navigating system constraints. These differences may stem from power dynamics, where HCPs control access to information and decision‐making, and from time pressures that force prioritisation of efficiency over relational care. Such dynamics risk creating communication that is technically correct but misaligned with what patients perceive as most meaningful. Addressing this gap requires strategies that support HCPs' management of workload while preserving the relational, person‐centred aspects of care that build trust and enable partnership with patients, emphasising mutual respect and shared decision‐making.

## 5 Discussion

The aim was to explore and compare patients' and HCPs' experiences of communication during hospitalisation for neurological diseases inspired by a qualitative photo‐voice method. The findings revealed that the core of communication was connected care, supported by a shared understanding, a supporting environment and humanising care. Effective communication is essential for delivering high‐quality healthcare based on a strong mutually trusting relationship between HCPs and patients (Haverfield et al. 2020). This, in turn, enhances healthcare outcomes and increases patient satisfaction (Haverfield et al. 2020). In this study, connected care included three themes that relate closely to person‐centred care (PCC) and shared decision‐making (SDM) (Barry and Edgman‐Levitan 2012; McCormack and McCance 2016). PCC is a broad umbrella concept involving theoretical frameworks such as Person‐centred practice (McCormack and McCance 2016), Fundamentals of care (Kitson et al. 2010) and humanisation of care (Busch et al. 2019). In these models, individuals' values are central to the decision‐making process and care outcomes. PCC emphasises individualised, holistic treatment that respects patients' values, preferences and goals while ensuring they are active participants in care (Busch et al. 2019; Kitson et al. 2010; McCormack and McCance 2016). Patients' accounts highlighted autonomy as a foundational principle in PCC (McCormack and McCance 2016). Their wish to be seen as active decision‐makers and to receive individual attention reflects the PCC value of respecting personal preferences and recognising the individual. Studies confirm that autonomy and patient involvement are essential for engagement, satisfaction and emotional well‐being (Doherty et al. 2020; Olchowska‐Kotala et al. 2023).

Our findings show that Guided alignment reflects both SDM and PCC principles, where information exchange, shared understanding and partnership support patient involvement (Barry and Edgman‐Levitan 2012; McCormack and McCance 2016). Changing environment corresponds to supportive contextual conditions of PCC, illustrating how organisational and structural settings shape opportunities for shared decision‐making and person‐centred practice (McCormack and McCance 2016). Human before patient highlights the PCC dimension of dignity, emotional support and recognition of the person beyond disease. McCormack and McCance 2016 emphasised the importance of ‘*sympathetic presence*’ and supportive environments in PCC (McCormack and McCance 2016); Deuling et al. (2025) demonstrated how leadership and care culture influence implementation (Deuling et al. 2025) and Rutherford et al. (2025) argued for strengthening PCC through measurable patient‐reported outcomes (Rutherford et al. 2025). Collectively, these themes resonate with McCormack and McCance's (2016) PCC domains: Alignment (shared decision‐making), environment (care setting) and humanity (dignity) (McCormack and McCance 2016). They are further reinforced by empirical evidence highlighting the role of leadership, care culture and outcomes measurement in sustaining PCC (Deuling et al. 2025; Rutherford et al. 2025). Together, they demonstrate how PCC and SDM shaped communication in neurological care, reinforcing their relevance for promoting autonomy, participation and connected care (Kwame and Petrucka 2021; Van Der Ploeg‐Dorhout et al. 2024).

Patients' narratives also played a central role in creating a shared understanding. Narrative medicine provides a framework that values personal stories as essential to grasping the lived experience of health and illness (Polkinghorne 1988). HCPs emphasised the importance of story‐based understanding in the diagnostic process, which is particularly relevant in neurological care, where up to 90% of hospital admissions in Denmark are acute and involve new or worsening symptoms (Sundhedsstyrelsen 2025). Narratives do more than organise events chronologically. They help reveal causal connections, guide clinical reasoning and uncover deeper meaning (Kleinman 1988; Polkinghorne 1988). This structure enables HCPs to integrate symptoms, events and patient experiences into a coherent whole, which is vital for early detection and effective care. Through shared storytelling, trust, empathy and emotional insight are built, offering a richer understanding than structured questioning alone (Kleinman 1988). It allows HCPs to relate to patients as whole persons rather than clinical cases, thereby promoting more compassionate, reflective and holistic care (Polkinghorne 1988). However, quantitative studies have reported that 43% of patients misunderstood their diagnoses, pointing to risks of fragmented or unclear communication (Olson and Windish 2010). Our participants, however, valued continuity as a way to maintain trust and relational care, underscoring the need to balance personal stories with accurate, evidence‐based information. Therefore, narrative has an ethical implication in healthcare, as it calls on HCPs to combine clinical facts with meaning (Kleinman 1988). However, the findings revealed barriers: HCPs spoke of time pressure, structural limitations and a lack of systemic focus on PCC, which made narratives difficult to integrate into daily practice.

This study found systemic barriers such as staff shortages, overcrowding and high patient loads, which created pressure and limited time for person‐centred dialogue and SDM. Patients sought recognition of their lived experiences, while HCPs were constrained by routines, hierarchies and time pressure. Systemic barriers are known as factors that shift priorities towards efficiency rather than relational, person‐centred care, undermining patient autonomy and the care relationship (Adhikari et al. 2025; Kwame and Petrucka 2021). As this study shows, these systemic barriers reinforce the risk that communication becomes task‐oriented rather than relational, compromising both patient satisfaction, autonomy and care quality.

Engaging patients in their healthcare through shared decision‐making is a fundamental principle of patient autonomy and PCC (Barry and Edgman‐Levitan 2012; Kwame and Petrucka 2021; McCormack and McCance 2016; van der Horst et al. 2023). PCC is considered the best practice in complex conditions such as neurological disease, where the clinical focus often is on symptom management and quality of life (Sundhedsstyrelsen 2025). Patients face complex treatment plans and uncertainty, making transparent communication and collaboration imperative (Sundhedsstyrelsen 2025). Patients with neurological disease(s) may also struggle with memory, processing speed and information handling (Foronda et al. 2016; Paynter et al. 2019), requiring adaptations such as visual aids and decision‐supportive tools, involving relatives and training HCPs in communication techniques and SDM techniques (Cypher 2019; Kwame and Petrucka 2021). SDM can increase patient trust, understanding and satisfaction with care (National Institute for Healthcare and Care Excellence (NICE) 2021). Although SDM can be time‐consuming, evidence shows that improving comprehension does not necessarily increase provider workload (Van Der Ploeg‐Dorhout et al. 2024).

### 5.1 Methodological Considerations

The strength of this study was to incorporate patients and HCPs' experiences combined with photographs to support verbal narratives. This approach offered a holistic and nuanced understanding of communication dynamics in neurological care (Catalani and Minkler 2010). Conducting interviews during hospital admission and using photos as elicitation tools allowed participants, particularly those with cognitive or speech impairments, to express their experiences in concrete ways. This contributed to important insights into communication challenges in this setting.

One of the limitations of the study was that interviews with HCPs were conducted during working hours. This timing, while practical, meant that some participants appeared time‐pressured and may have provided brief responses. The clinical context may have limited their ability to engage more deeply with the interview questions or to elaborate on their experiences, potentially affecting the richness of the data (Malterud 2011). Another methodological limitation relates to conducting the study in a familiar setting. Familiarity with the study context may enhance participants' willingness to share their experiences and facilitate access to the research field (Malterud 2001). Two authors had clinical experience in neurology, and three were familiar with the department in which the study was conducted. This background facilitated access to the field, trust‐building with participants and sensitivity to contextual nuances. However, familiarity can create a ‘blind spot’ that may mask unfamiliar or problematic aspects of the setting, potentially influencing both data collection and interpretation (Berger 2015). To address this, we engaged in reflexive dialogue, maintained field notes, and involved a nonaffiliated researcher with a background in sociology in the analysis to challenge internal interpretations, critically examine our own preconceptions and assumptions and broaden our analytical perspective (Braun and Clarke 2021; Malterud 2001). Further limitations include the single‐centre design, which may restrict the transferability of findings to other clinical settings (Guba and Lincoln 2005; Malterud 2001). For patients with cognitive impairments, recall bias is a potential concern, as they may have difficulty accurately remembering experiences (Cochrane 2022; Paynter et al. 2019). Although this was partly addressed by interviewing participants during hospitalisation and using visual cues, the influence of fatigue, stress or cognitive deficits on data quality cannot be fully ruled out (Cochrane 2022; Harper 2012; Wang and Burris 1997). Despite these limitations, several strategies were employed to enhance the study's trustworthiness. The interview guide was grounded in empirical insights, contributing to the robustness of data collection (Guba and Lincoln 2005; Malterud 2001). During interviews, the interviewer actively sought clarification to ensure an accurate understanding. Credibility was supported by presenting illustrative participant quotes for each theme and by employing investigator triangulation in coding and analysis (Guba and Lincoln 2005; Malterud 2001). To enhance confirmability, the research team engaged in thorough discussions of the findings, reflected on preconceptions and considered alternative interpretations before reaching a shared understanding (Guba and Lincoln 2005; Malterud 2001).

### 5.2 Implication for Policy and Practice

This study is relevant to clinical practice, as it enables healthcare professionals to improve communication strategies by prioritising the implementation of person‐centred care. By integrating guided alignment, environmental considerations and individualised care priorities, effective communication can contribute to connected care, with the potential to enhance patient engagement and improve health outcomes. These findings provide evidence‐based insights into the importance of rethinking the structure and support of communication in clinical settings. Instead of isolated initiatives, communication should be approached as a continuous, integrated process embedded in the culture of care, because effective communication is more than tools. It depends on reflection, teamwork and shared responsibility. Creating space for regular communicative supervision and reflective practice sessions can strengthen team cohesion and enable staff to develop more responsive person‐centred communication styles. Concrete strategies may include role‐playing exercises, simulation‐based training including aphasia simulation exercises or the use of visual aids to support shared decision‐making. Introducing dedicated roles, such as communication champions or patient pathway coordinators, may promote continuity and sustained focus on the patient's voice across care. Moreover, involving patients and relatives in codesigning care processes and developing information materials or decision aids can ensure relevance and accessibility. On a policy level, these findings emphasise the importance of institutional support for patient‐centred communication. This includes investing in staff training, interdisciplinary collaboration and the development of mandatory adaptable communication guidelines that can be tailored to local contexts.

## 6 Conclusions

This study highlights the crucial role of effective communication in person‐centred neurological care, emphasising the need for alignment between healthcare professionals and patients despite cognitive and motor challenges. By identifying key themes (guided alignment, changing environment and human before patient), the findings illustrate how structured communication strategies can provide a shared understanding, enhance patient engagement and improve care experiences. The ‘aquarium’ metaphor, based on participants' photos, reflects their experience of being visible yet separated, observed but not engaged. It highlights the tension between institutional routines and the wish for relational, person‐centred care. This emphasises how balanced communication enables both patients and professionals to thrive, especially when cognitive and motor challenges, including the surrounding environment, shape interactions. Addressing these communication barriers through training, shared decision‐making frameworks and interdisciplinary collaboration can improve patient engagement, reduce misunderstandings and enhance neurological care outcomes. Future research should not only evaluate but also codesign and implement targeted communication strategies, in close collaboration with patients, relatives and interdisciplinary teams, and evaluate communication changes after discharge.

## Author Contributions

These authors contributed differently to this work. J.F.J., L.G.H. and P.W.B. made substantial contributions to conception, design and data acquisition. J.F.J. and C.F.J. were responsible for the initial analysis and interpretation of data. J.F.J. and C.F.J. were involved in drafting the manuscript. L.G.H. and P.W.B. revised it critically for important intellectual content. J.F.J., C.F.J., L.G.H. and P.W.B. have all given final approval of the version to be published. Each author should have participated sufficiently in the work to take public responsibility for appropriate portions of the content. J.F.J., C.F.J., L.G.H. and P.W.B. agreed to be accountable for all aspects of the work in ensuring that questions related to the accuracy or integrity of any part of the work are appropriately investigated and resolved.

## Ethics Statement

The National Committee on Health Research Ethics (not subject to Ethical committee review: no. EMN‐2023‐02212) and the Danish Data Protection Agency (EMN‐2023‐04115) approved the study. All participants gave written consent to participate in this study.

## Conflicts of Interest

The authors declare no conflicts of interest.

## Supporting information


**Table S1:** Examples of the analysis supplemented with photographs.

**Supporting Information file 1** Consolidated criteria for reporting qualitative research, the COREQ checklist.

## Data Availability

The data that support the findings of this study are available from the corresponding author upon reasonable request.
