# Supplementary material for: Communication Between Patients and Healthcare Professionals in Neurological Hospitalisation: A Qualitative Photo‐Voice Study
Source: J Clin Nurs. 2025 Oct 9;35(4):1752–65. doi: 10.1111/jocn.70122 (PMC12964512; doi:10.1111/jocn.70122)
Supplement: Supplementary file 1 — Table S1: Examples of the analysis supplemented with photographs. [file JOCN-35-1752-s001.docx]

**Supplementary materials**

**Table S1: Example of the analysis supplemented with photographs.**

An example to illustrate how the joint analysis was conducted and how photographs supported participants’ statements based on Theme III: Human before patient – seem with patients’ perspectives

| Selected Quotes | Photographs | Initial codes | Developed Codes | Sub-Themes | Theme |
| --- | --- | --- | --- | --- | --- |
| I prefer to be clean. But I don’t want to be a burden (P6) | 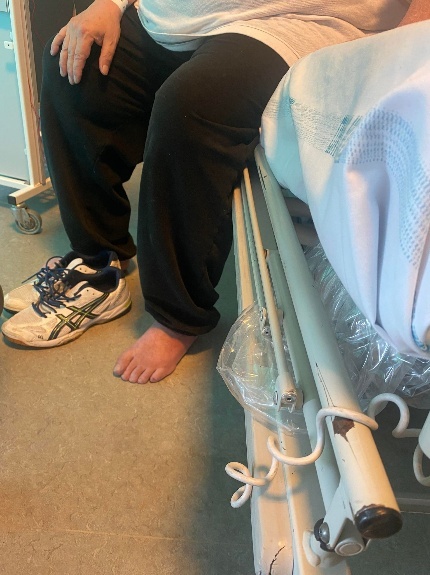  [Misses socks to wear] | Presenting myself nicely | Individuality | Knowing me | Human before patient |
| Now, I am a special type, I’m an officer, so I’ll make sure to get my way – also in my way (P10) | 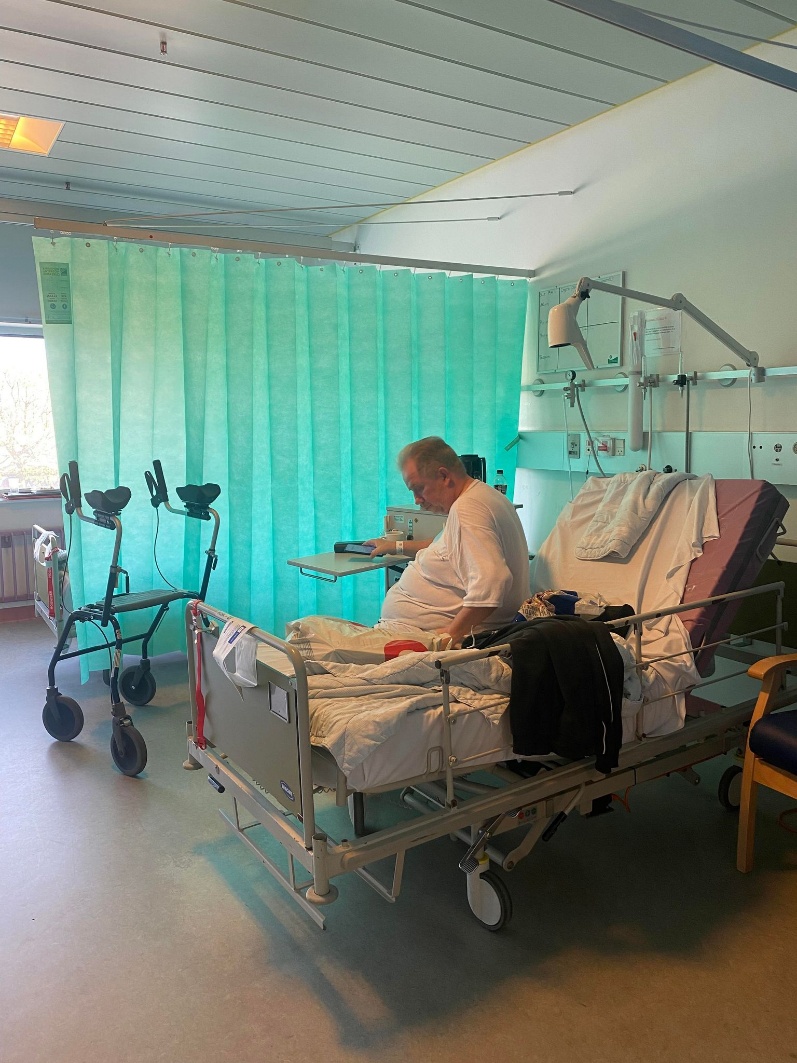 | A special person, who wants it my Way |  |  |  |
| I have my brain calendar for all healthcare appointments (P10)  An old-fashioned spiral calendar. I’m so old that I use pen and paper. I also have to use the end of a pen on my phone, because I can’t hit the keys with my fingers here (P2) | 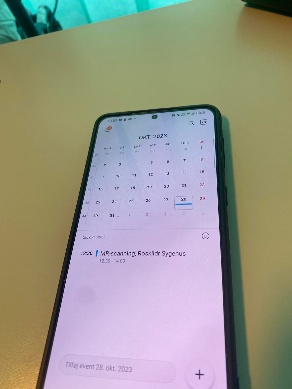  [Calendar]  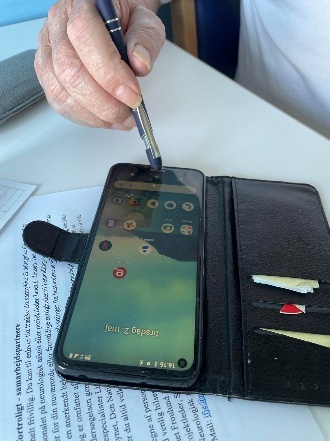  [Pen and paper or note on my phone] | Assisting communication aids for memory, coordination, and verbalization | Encouraging active participation |  |  |
| Maybe it’s a symptom too; it should have been loud enough, otherwise I wouldn’t have noticed it, and there may have been many symptoms before that I didn’t notice (P3) | 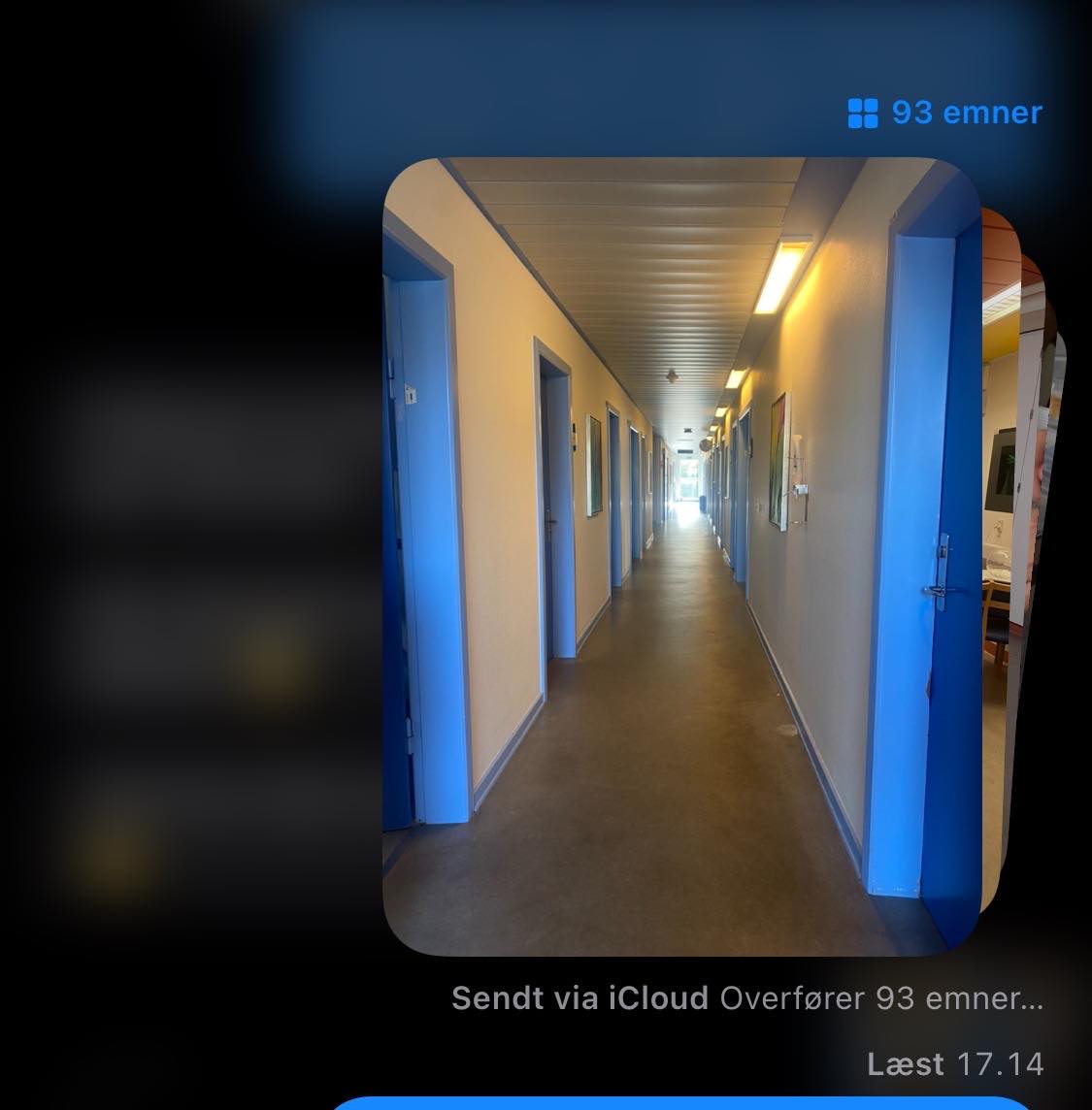  [A calm hallway was photographed with ironic intent to illustrate loud cognitive symptoms] | Recognition of symptoms | Cognitive-interfering symptoms |  |  |
| I live a lot in my head… But I can also talk to them (nurses) about more personal things, and they share their advice and so on. They really listen. It’s not always possible to talk to your family about such things regarding your illness and what’s going on in your head, because it also affects them. So, it’s easier to come here, and it’s nice that they listen to me and actually spend that extra time on it (P4) | 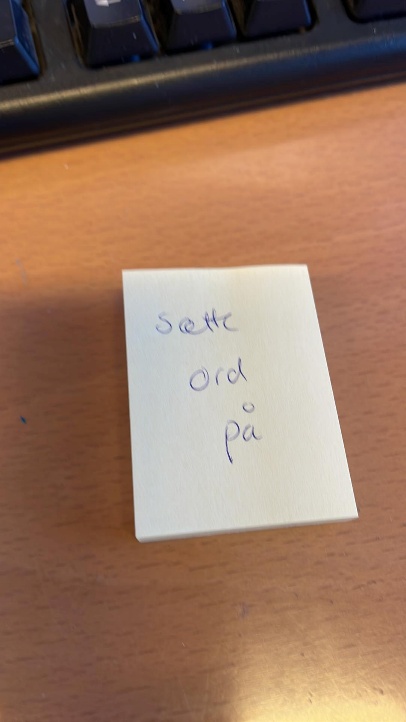  [Put (thoughts) into words] | Verbalizing what’s on my mind | Being seen | Respect and dignity |  |
| I tease the doctor a bit and push the chair over to the bed. It’s best to be able to look at each other in the eyes (P8) | 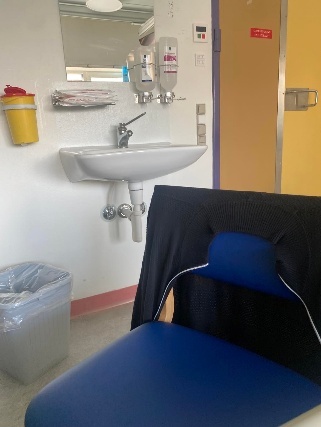 | At eye level | Seen and respected |  |  |
| The paper (the pamphlet) is fine, but I’ll read it later, so much else is happening here (P2)  My health platform has many options in some way. I think it can quickly become a little confusing (P9)  Time passes slowly; I like to read about what’s happening in the world. It’s a good book (P8) | 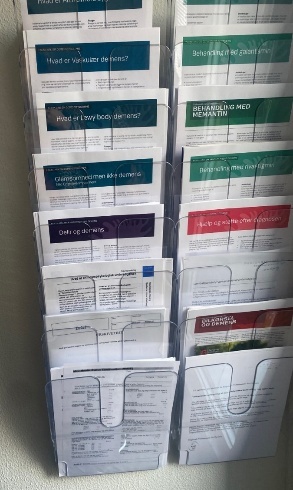  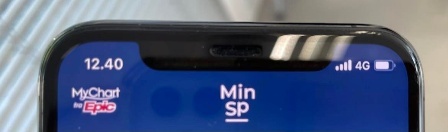  [Written and online information] 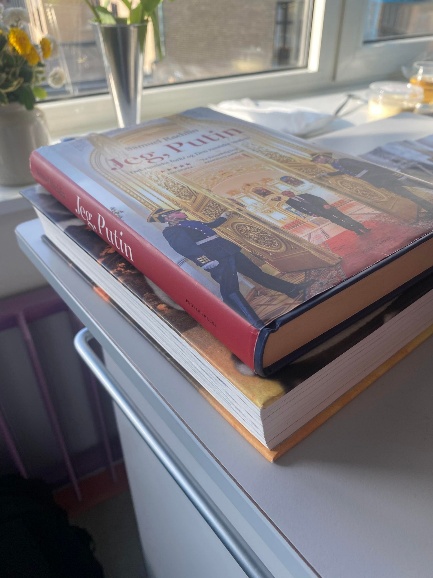  [Reading and keeping me updated] | Methods to understand | My values and preferences |  |  |
| It has probably been misunderstood, or at least not really heard (P9)  ……  I’m having an X-ray taken of my life. These are conversations with a senior physician that I remember as very competent and very direct. … It was very overwhelming to look my own life in the eye that way, but when I thought about the conversations, I could actually see the idea, just as he said (P11) | 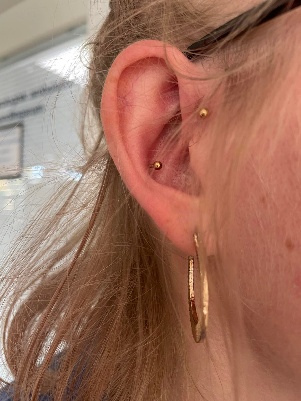 | Listen to the experts | Acknowledge others expertise | Trust |  |
| Well, a picture of this could be a smiling nurse – that makes me want to be happy. But with the medicine here, there is probably more than just medicine in the medicine you get here, that’s why I took this picture (P4) | 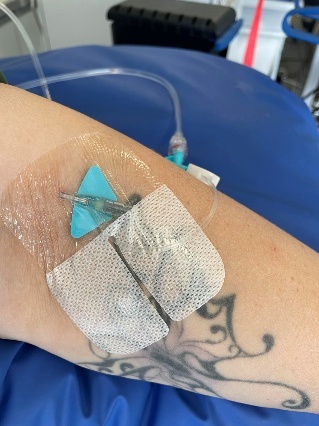 | More than medicine, I am in their hands | The human side |  |  |
| I spend a lot of time looking for this cord bell, I really do. Sometimes I get hold of it, but I’ve pulled too hard and ripped it out. So, I’m actually quite worried about that cord (P7) | 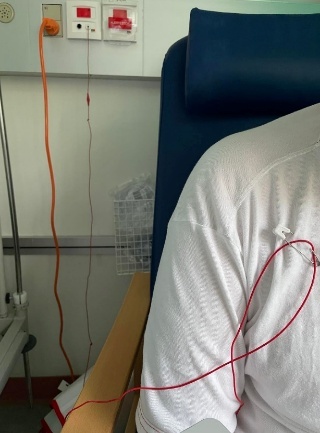 | Cord bell to contact staff | Feeling safe and having support around | Lifelines |  |
| My wife is worth gold; she makes me relax. She gives me peace (P8)  I was restless and confused (at admission). My daughter had the only sense of security in the situation (P12) | 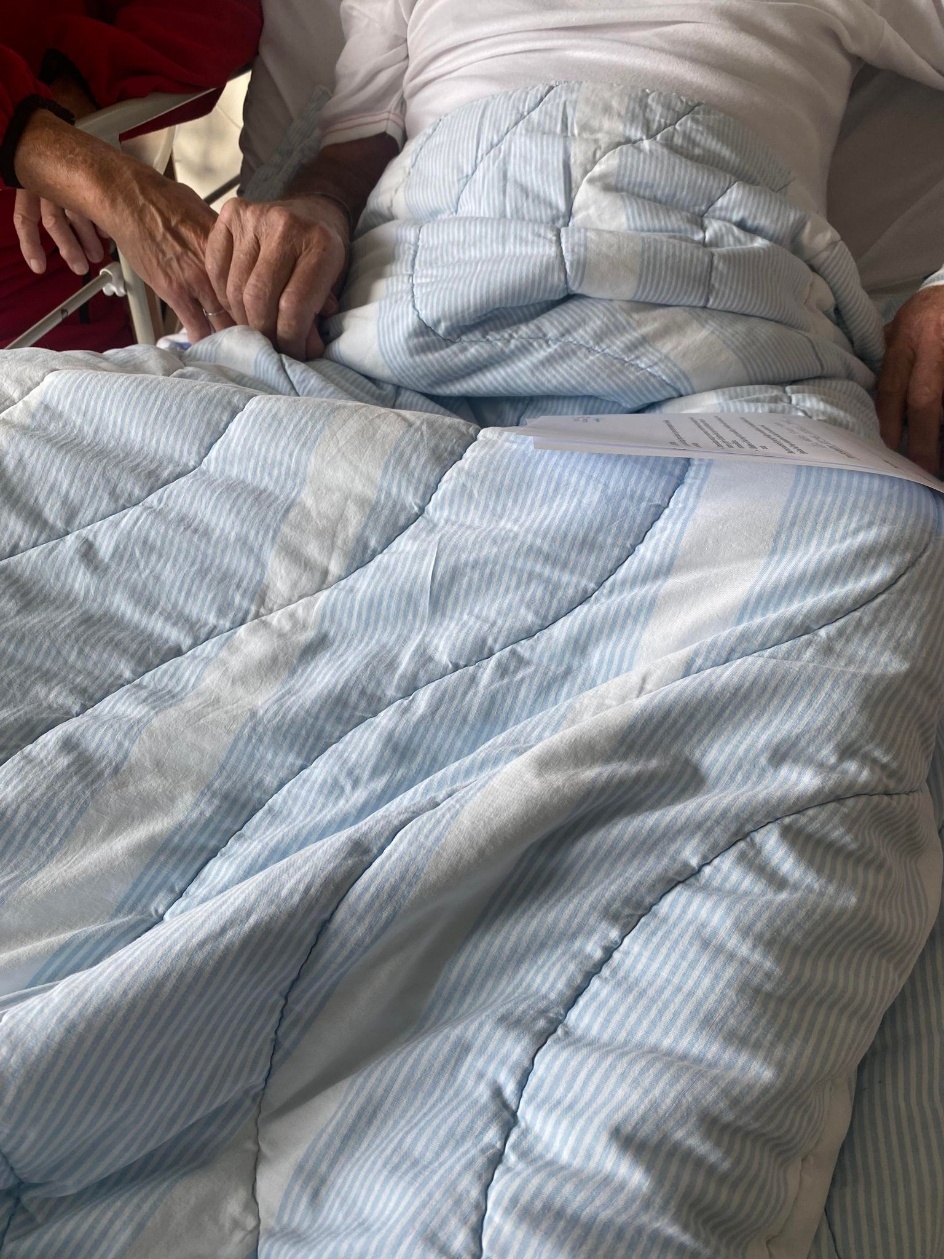  [Supported by family] | Breathing space |  |  |  |
| I’ve lost some expensive sunglasses at the hospital. So, I take care of my things, because I don’t want to lose those too (P7) | 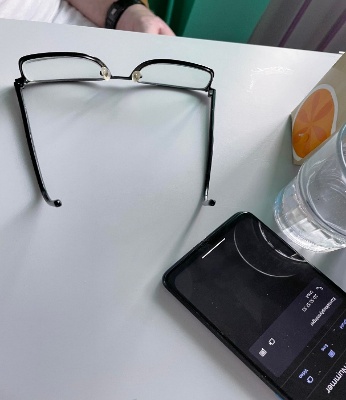 [Having my glasses and phone nearby] | Calms me down to have my things |  |  |  |
| I’ve talked a lot with him over there (fellow patient in the room), and I like to talk a lot, maybe because I’m a pedagogue. … I’ve tried being in a single room, but I prefer two- or four-bed rooms, because having each other makes you feel less alone (P7) | 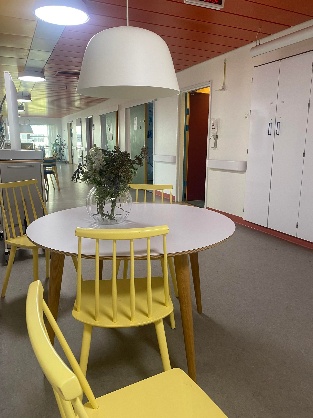  [Peers to talk to] | Peers are my fellow patients |  |  |  |
