# Supplementary material for: Communication Between Patients and Healthcare Professionals in Neurological Hospitalisation: A Qualitative Photo‐Voice Study
Source: J Clin Nurs. 2025 Oct 9;35(4):1752–65. doi: 10.1111/jocn.70122 (PMC12964512; doi:10.1111/jocn.70122)
Supplement: Supplementary file 2 — Supporting Information file 1 Consolidated criteria for reporting qualitative research, the COREQ checklist. [file JOCN-35-1752-s002.docx]

# **Supplementary materials**

**Table 1 Consolidated criteria for reporting qualitative studies (COREQ): 32-item checklist**

| **Item** | **Guide questions/description** | **Page #** |
| --- | --- | --- |
| **Domain 1: Research team and reflexivity** |  |  |
| **Personal Characteristics** |  |  |
| 1. Interviewer/facilitator | Which author/s conducted the interview or focus group? | 10 |
| 2. Credentials | What were the researcher’s credentials? E.g. PhD, MD | Title page, 13 |
| 3. Occupation | What was their occupation at the time of the study? | Title page |
| 4. Gender | Was the researcher male or female? | Title page |
| 5. Experience and training | What experience or training did the researcher have? | Title page, 10, 29 |
| **Relationship with participants** |  |  |
| 6. Relationship established | Was a relationship established prior to study commencement? | Title page, 10-11, 29 |
| 7. Participant knowledge of the interviewer | What did the participants know about the researcher? e.g. personal goals, reasons for doing the research | 10-11, 29 |
| 8. Interviewer characteristics | What characteristics were reported about the interviewer/facilitator? e.g. Bias, assumptions, reasons and interests in the research topic | 9, 10, 29 |
| **Domain 2: study design** |  |  |
| **Theoretical framework** |  |  |
| 9. Methodological orientation and Theory | What methodological orientation was stated to underpin the study? e.g. grounded theory, discourse analysis, ethnography, phenomenology, content analysis | 7-8, 11 |
| **Participant selection** |  |  |
| 10. Sampling | How were participants selected? e.g. purposive, convenience, consecutive, snowball | 8-9 |
| 11. Method of approach | How were participants approached? e.g. face-to-face, telephone, mail, email | 9 |
| 12. Sample size | How many participants were in the study? | Figure 1, 8, 13 |
| 13. Non-participation | How many people refused to participate or dropped out? Reasons? | 10 |
| **Setting** |  |  |
| 14. Setting of data collection | Where was the data collected? e.g. home, clinic, workplace | 8-10 |
| 15. Presence of non-participants | Was anyone else present besides the participants and researchers? | 10 |
| 16. Description of sample | What are important characteristics of the sample? e.g. demographic data, date | 13  table 1 & 2 |
| **Data collection** |  |  |
| 17. Interview guide | Were questions, prompts, guides provided by the authors? Was it pilot tested? | 10 |
| 18. Repeat interviews | Were repeat interviews carried out? If yes, how many? | no |
| 19. Audio/visual recording | Did the research use audio or visual recording to collect the data? | 10 |
| 20. Field notes | Were field notes made during and/or after the interview or focus group? | 10, 11, 13, 29  Reflection notes |
| 21. Duration | What was the duration of the interviews or focus group? | 10 |
| 22. Data saturation | Was data saturation discussed? | 10 |
| 23. Transcripts returned | Were transcripts returned to participants for comment and/or correction? | Fous group discussion were, 10 |
| **Domain 3: analysis and findings** |  |  |
| **Data analysis** |  |  |
| 24. Number of data coders | How many data coders coded the data? | 11 |
| 25. Description of the coding tree | Did authors provide a description of the coding tree? | 16  Figure 2 |
| 26. Derivation of themes | Were themes identified in advance or derived from the data? | 11 |
| 27. Software | What software, if applicable, was used to manage the data? | 13 |
| 28. Participant checking | Did participants provide feedback on the findings? | No  Staff has |
| **Reporting** |  |  |
| 29. Quotations presented | Were participant quotations presented to illustrate the themes / findings? Was each  quotation identified? e.g. participant number | 16-24  In texts, yes |
| 30. Data and findings consistent | Was there consistency between the data presented and the findings? | 16-24, figure 2  Yes |
| 31. Clarity of major themes | Were major themes clearly presented in the findings? | 15, figure 2  Yes |
| 32. Clarity of minor themes | Is there a description of diverse cases or discussion of minor themes? | 16-24 |
